# Rehabilitation effects of land and water-based aerobic exercise on lung function, dyspnea, and exercise capacity in patients with chronic obstructive pulmonary disease: A systematic review and meta-analysis

**DOI:** 10.1097/MD.0000000000026976

**Published:** 2021-08-20

**Authors:** Haixia Chen, Peijun Li, Ning Li, Zhengrong Wang, Weibing Wu, Jihong Wang

**Affiliations:** a School of Physical Education and Training, Shanghai University of Sport, Shanghai, China; b Department of Sports Rehabilitation, Shanghai University of Sport, Shanghai, China.

**Keywords:** aerobic exercise, chronic obstructive pulmonary disease, exercise therapy, water

## Abstract

**Background:**

We sought to synthesize the evidence about aerobic exercise intervention during pulmonary rehabilitation, and to further explore the difference in rehabilitation effects between water and land-based aerobic exercise. This review's purpose is to provide a basis by which practitioners and therapists can select and create appropriate therapeutic programs.

**Methods:**

Data of randomized and quasi-randomized controlled trials comparing training group (TG, aerobic exercise in water or land) and control group (CG, usual care) in chronic obstructive pulmonary disease (COPD) patients (January 1, 2000–December 28, 2019) were obtained from the Cochrane Library, PubMed, Embase, China National Knowledge Infrastructure, and Wanfang databases. Two researchers independently reviewed the literature, extracted the data, and evaluated the quality of the literature. Review Manager software (Rev Man 5.3; Cochrane, London, UK) was used for meta-analysis. The rehabilitation effect of water- or land based aerobic exercise was evaluated by subgroup analysis. The proposed systematic review details were registered in PROSPERO (CRD 42020168331).

**Results:**

Eighteen studies (1311 cases of COPD) were included. Meta-analysis results show that compared with the control group, the dyspnea level and functional and endurance exercise capacity in COPD patients were significantly improved after aerobic exercise (*P* < .05), but there was no significant change in lung function (*P* > .05). Compared with land-based aerobic exercise, water-based aerobic exercise significantly improved the endurance exercise capacity in COPD patients (mean difference [MD]: 270.18, 95% CI: 74.61–465.75).

**Conclusion:**

Medium to high-quality evidence shows that aerobic exercise can effectively improve dyspnea and exercise capacity in COPD patients. Compared with land-based aerobic exercise, water-based aerobic exercise had a significant additional effect in improving the endurance exercise capacity of COPD patients.

## 1 Introduction

Chronic obstructive pulmonary disease (COPD) is a common, preventable, and treatable chronic respiratory disease characterized by restricted airflow caused by abnormal airways and/or alveoli.^[1]^ Epidemiological data show that the prevalence of COPD in people over the age of 40 has reached 13.7%.^[2]^ Nearly three billion people worldwide are at risk of COPD,^[3]^ with >5.4 million deaths a year from COPD and related diseases by 2060.^[4]^ As the disease progresses, patients often undergo frequent outpatient or inpatient treatment due to dyspnea, exercise intolerance, and decreased levels of daily living activities, eventually leading to a decline in quality of life, poor prognosis, and premature death.^[5]^ Although evidence-based drug therapy can alleviate dyspnea in COPD patients, a 2018 Lancet study^[2]^ and the 2020 GOLD guidelines^[6]^ still emphasize the importance of pulmonary rehabilitation (PR) in COPD.

PR is a personalized, multi-disciplinary, evidence-based medicine, and it is a comprehensive non-drug intervention program designed for COPD patients. It is an essential element of a comprehensive nursing strategy for COPD. As the cornerstone of PR, aerobic exercise can improve maximum exercise performance in COPD patients (moderate–severe), enhance exercise tolerance and physiological adaptation, and facilitate the self-regulation of heart rate.^[7,8]^ In recent years, aerobic exercise programs for COPD patients have been continually enriched (both land-based aerobic exercise and water-based aerobic exercise). The main roles of aerobic exercise are the optimization of walking-related skeletal muscle function, the improvement of, cardiopulmonary fitness, exercise capacity, and physical activity.^[8,9–11]^ A rich variety of aerobic exercise programs presents more possibilities and options to patients seeking PR, however, these programs also broaden the methodologies used for aerobic exercise intervention, which leads to variation in the rehabilitation effects of aerobic exercise. Therefore, the effectiveness of these aerobic exercise programs requires further verification.

Studies have shown that in land-based aerobic exercise intervention, chronic joint pain,^[12]^ skeletal muscle dysfunction,^[13]^ and imbalance^[14]^ patients have poor rehabilitation effects. Patients often fail to adhere for a long period time due to lack of interest,^[15]^ and as their disease condition worsens, they are unable to complete the established intervention program. A meta-analysis found that there are additional advantages in water,^[16]^ which may be due to the fact that the water environment has physical characteristics such as temperature, hydrostatic pressure, buoyancy, and resistance, which play a positive role in relieving pain and reducing joint load for the lower extremities. Due to the load effect of aerobic exercise in water on respiratory muscles, it may have a significant positive effect on improving lung function,^[17]^ respiratory muscle strength,^[18]^ and dyspnea^[19]^ in COPD patients. However, the study found that there was no difference in changes to lung function or exercise capacity between land and water-based exercise, and there was still insufficient evidence to support the conclusion that either land or water-based exercise led to significant improvement in lung function and exercise capacity.

This review aimed to quantitatively evaluates the effects of aerobic exercise on lung function, dyspnea, and exercise capacity in COPD patients when used in water or on land. This can provide a basis that will help practitioners and therapists selecting and structuring appropriate therapeutic programs to achieve improvement in all aspects of COPD, and promote an overall therapeutic effect.

## 2 Methods

This systematic review is reported in accordance with the Preferred Reporting Items for Systematic Reviews and Meta-Analyses (PRISMA) guidelines. The proposed system evaluation details have been registered in PROSPERO (CRD42018094172). Our manuscript is meta-analysis, so ethical review is unnecessary.

### 2.1 Search strategy

The Cochrane Library, PubMed, Embase, China National Knowledge Infrastructure, and Wanfang databases were searched for randomized controlled trials and quasi-randomized controlled trials of land and or water aerobic exercise for COPD patients from January 1, 2000 to December 31, 2019, regardless of publication language. When formulating the retrieval strategy, 3 groups of subject words and related keywords that included participants, interventions, and experimental design were used. The primary search words are shown in Table 1. To complement these procedures, references included in the studies, related system reviews, and meta-analyses were hand searched to identify other studies that could be eligible.

**Table 1:** Main search terms.

Subject	Key words
Pulmonary disease, chronic obstructive; lung diseases, obstructive	COPD; Chronic Obstructive Lung Disease; Chronic Obstructive Airway Disease; Chronic Airflow Obstructions
Exercise; aerobic-exercise; water aerobics; water sport	Water-based exercise; Water-based sports; Exercises, Aerobic; Aerobic Exercises; Aerobic training; Endurance Training; walking
Randomized controlled trial	Randomized; placebo

### 2.2 Inclusion criteria

All the included publications were randomized controlled studies or quasi-randomized controlled studies, the study subjects were patients with stable COPD, the experimental group received aerobic exercise intervention on land (LG) or in water (WG) along with usual care, while the control group (CG) only received usual care, the specific forms of aerobic exercise could be carried out in water or on land, and at least one outcome index was evaluated, and these included different parameters to evaluate lung function, namely, forced expiratory volume in the first second in percent predicted values (FEV1%pred), ratio of forced expiratory volume in the first second to forced vital capacity (FEV1/FVC%); exercise capacity, 6-minute walking test (6MWT), incremental shuttle walk test (ISWT), endurance shuttle walk test (ESWT); dyspnea (Borg); peripheral muscle strength (upper limb muscle strength [ULMS]: biceps brachii, lower limb muscle strength [LLMS]: quadriceps femoris). Among them, dyspnea and peripheral muscle strength are secondary outcomes.

### 2.3 Exclusion criteria

Patients with acute exacerbation of COPD, outcomes that did not meet the requirements, data could not be extracted, outcomes did not meet the requirements, and non-randomized controlled trials.

### 2.4 Quality evaluation

The PEDro score was used to evaluate the quality of the articles that included random allocation; concealed allocation; baseline comparability; blind subjects, therapists, and assessors; adequate follow-up; intention-to-treat analysis; between-group comparisons; and point estimates and variability. Items are rated yes or no (1 or 0) according to whether the criterion is satisfied in the study. A total PEDro score is achieved by adding the ratings of items 2 to 11 for a combined total score between 0 and 10. Higher scores indicate superior methodological quality. Study quality did not affect eligibility for inclusion within the meta-analysis, as detailed in the resources (https://www.pedro.org.au).

### 2.5 Data extraction

Two researchers evaluated the title or abstract of each article separately. At least one of the researchers had to consider that the study met the inclusion criteria to be included in the first selection for a full evaluation. The 2 researchers then independently evaluated the selected articles to determine whether to include or exclude them from the study. When disagreement occurred, a third researcher participated in the discussion to reach a final consensus. Finally, for studies that met the inclusion criteria, the study characteristics and required data were extracted. The extracted contents included the participant sample size, participant age, sex ratio, degree of airflow restriction (FEV1% predicted), intervention programs (such as the training format, content, exercise intensity, intervention cycle), and outcomes.

### 2.6 Data analysis

Review Manager software (Rev Man 5.3, Cochrane, London, UK) was used for the meta-analysis. For binary variables, the odds ratio was used as the effect scale index. For the continuous variables, the mean difference (MD) or standardized mean difference (SMD) and a 95% confidence interval (CI) were used. The same measurement method and units were used to report the research results using MD, and different measurement methods or units were used to report research results using SMD. If *P* < .05, a significant difference existed. Heterogeneity between studies was assessed using *I*^2^ statistics, and *I*^2^ values <25%, 25% to 50%, and >50% were considered to represent small, medium, and large heterogeneity, respectively. In this meta-analysis, we selected the random effects model for the combined analysis.

## 3 Results

### 3.1 Search results

**Figure 1:**
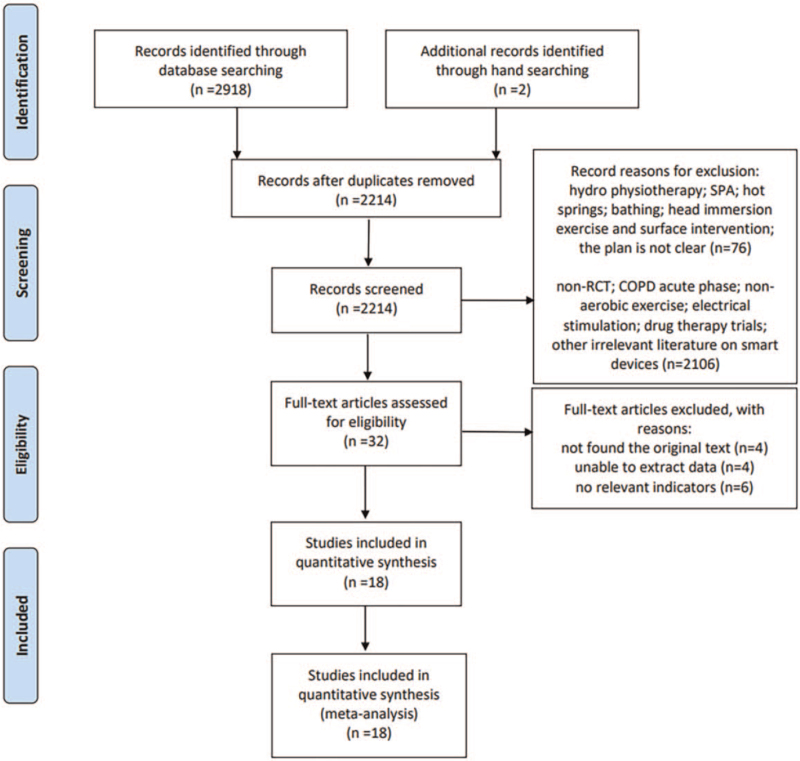
Flowchart for the identification of studies included in the meta-analysis.

A database search was performed to obtain 2918 studies. After deleting 706 repetitive studies, reading questions, abstracts, and excluding the full text, 18 studies were subsequently selected for the final analysis (Fig. 1).

### 3.2 Study characteristics

Eighteen studies involving 1311 patients were included, of which 651 (50%) performed aerobic exercise on land, 177 (13%) performed aerobic exercise in water, and 483 (37%) received usual care. The participants were an average age of 58 to 75 years, with moderate to severe disease. Eighteen studies were included, 9 studies^[20–28]^ explored the intervention effects of aerobic exercise on land, 2 studies^[29,30]^ explored the effects of aerobic exercise interventions in the water environment, 3 studies^[31–33]^ compared the differences between the effects of aerobic exercise interventions performed on land and water environments, and 4 studies^[34–37]^ compared the intervention effects between aerobic exercise on land, aerobic exercise in water, and the CG. In all studies, the intensity of walking and running was 70% to 80% of the average 6MWT speed or Borg scale of 4 to 6 points, and cycling exercise at 60% to 70% of the individual's maximum speed or Borg scale of 4 to 6 points, upper and lower limb strength exercise is a low-intensity and high-repetition exercise based on physiological tolerance. The intervention cycle focused on 6 to 12 weeks (2–5 times/wk). The water exercise was performed in a swimming pool with a water temperature of 32 to 34 °C. The specific forms and movements can also be applied to land-based aerobic exercise. In the comparative study of water and land, most of the aerobic exercise that took place in water used a floating device to increase the resistance, or reduce the initial load to reduce the resistance, and took into account the respiratory muscle and auxiliary muscle movement that may be brought about by higher thoracic and abdominal pressure during the exercise, to enhance the comparability of water and land-based exercise intensity. Table 2 details the included study participants, intervention programs, and outcomes.

**Table 2:** Included research characteristics.

Study	WG	LG	CG	Comeout/Index	Result
Bestall 2003^[20]^	—	n = 23; yr: 68.2±8.4; FEV1%pred: 37 ± 11; Intervention: ET (walking/cycling); ST; Intensity: Borg 3–4; Period: 2T/W, 8W; follow-1Y (3T/W, T/60 mins)	n = 21; yr: 69.2 ± 6.3 FEV1%pred: 38 ± 12 routine nursing	ISWT; ESWT	LG: ISWT^a^
Wadell 2004^[36]^	n = 15; yr: 65 ± 4; f/m: 11/4; FEV1%pred: 56 ± 11 water temperature: 33–34 °C; Intervention: same as LG	n = 15, yr: 65 ± 7; f/m: 10/5; FEV1%pred: 53 ± 12; Intervention: 9 min WU; 4 min ET; 3 min ST, 12 min CD; Intensity: Borg 5; Period: T/45 min; 3T/W; 12W	n = 13; yr: 63 ± 7; f/m: 6/7; FEV1%pred: 49 ± 12; routine nursing	ISWT; ESWT	LG:ISWT^b^; WG: ESWT^a^
Puente-Maestu 2006^[21]^	—	n = 28; yr: 62 ± 5; FEV1%pred: 46.8 ± 9.2; Intervention: 45 min ET; Intensity: 70%peak (cycling) Period: 4T/W, 6W	n = 20; yr: 61 ± 6 FEV1%pred: 47.1 ± 10.9 routine nursing	FEV1%pred; FEV1/FVC%; Borg	LG: FEV1%pred^b^, FEV1/FVC%^b^
Lotshaw 2007^[31]^	n = 20; yr: 65 ± 14; f/m: 12/8 FEV1%pred: 47.11 ± 17; Intervention: same as LG; ST's intensity (2 × 10)	n = 12; yr: 71 ± 7; f/m: 11/9; FEV1%pred: 44.44 ± 15.84; Intervention: 30 min ET (running/cycling); 30 min ST; Intensity: HR 60–80%, Borg 11–14; 6RM100%; Period: 6W/18T	—	6MWT; 6RM	LG: All the indicators^b^
Ozdemir 2010^[30]^	n = 25; yr: 60.9 ± 8.8; FEV1%pred: 54.5 ± 15.6; water temperature 32 °C; Intervention: 35 min ET; ST, Period: 3T/W, 4W	—	n = 25; yr: 64.1 ± 8.9; FEV1%pred:54.1 ± 20.2; routine nursing	FEV1%pred; 6MWT; Borg	WG: FEV1%pred^c^; 6MWT^b^
de Souto Araujo 2012^[34]^	n = 8; yr: 62.4 ± 9.9; f/m4/4; FEV1%pred: 43.9 ± 10.3; water temperature 32 ± 2 °C; Intervention: 15 min (aerobics); 2 min UAEx; 30 min (cycling); 15 min CD; Intensity: weight (sling) and diagonal movement, maximum individual load 50%↑, Borg5; Period: 3T/W; 8W	n = 13; yr: 56.9 ± 7.9; f/m5/8; FEV1%pred: 39.2 ± 11.4; Intervention: 15 min (aerobics); 2 min UAEx, 30 min (cycling); 15 min CD; Intensity: weight (sling) and diagonal movement, maximum individual load 50%↑, Borg5; Period: 3T/W; 8W	n = 11; yr: 71.1 ± 10.1; f/m3/8 FEV1%pred: 45.1 ± 12.6 routine nursing	FEV1%pred; FEV1/FVC%; 6MWT; Borg	TG: FEV1%pred^b^, FEV1/FVC%^b^, Dyspnea (WG/CG)6MWT^b^
Leug 2012^[27]^	—	n = 19; Intervention: 60 min ET (Tai chi wrist load 0.5–1.5 kg); Intensity: Borg3; Period: 2T/W, 12W	n = 19; routine nursing	ISWT; ESWT; Borg	LG: all the indicators^b^
Casey 2013^[22]^	—	n = 178, yr: 68.8 ± 10.2; f/m: 61/117; FEV1%pred: 57.6 ± 14.3; Intervention: 10 min WU; 20 min ET (walking); 3 min ST (8–10 × 3); 5 min CD; Intensity: Borg4; Period: 2T/W, 12–14W	n = 172; yr: 68.4 ± 10.3; f/m:66/106; FEV1%pred: 59.7 ± 13.8 routine nursing	ISWT	LG: ISWT^b^
McNamara2013^[37]^	n = 18; yr: 72 ± 10; f/m: 13/5; FEV1%pred: 60 ± 10; water temperature 34 °C; Intervention: similar to the LG	n = 20; yr: 73 ± 7; f/m: 10/10; FEV1%pred: 62 ± 15; Intervention: 8 min WU; 15–20 min (walking/cycling); 10 min ET (3–10); 2 min CD; Intensity: 80% 6MWT (walking); Borg3–5; Period: 3T/W; 8W	n = 15; yr: 70 ± 9; f/m: 8/7 FEV1%pred: 55 ± 20 routine nursing	6MWT; ISWT; ESWT; Borg	LG: 6MWT^b^, ISWT^c^, ESWT^b^, Dyspnea^b^ WG: 6MWT^b^, ISWT^b^, ESWT^b^ WG/LG; Dyspnea^c^,
Wootton 2014^[23]^	—	n = 95; yr: 69 ± 8; f/m: 39/56; FEV1%pred: 43 ± 15; Intervention: 30 min ET (walking); Intensity: 80% 6MWT (load↑) Borg 3–4; Period: 2–3T, 8–10W	n = 48; yr: 68 ± 9; f/m: 20/28; FEV1%pred: 43 ± 15; routine nursing	ISWT; ESWT; 6MWT; Borg	LG: ISWT^b^, ESWT^a^
Tsai 2017^[24]^	—	n = 19; yr: 73 ± 8; f/m: 7/12; FEV1%pred: 60 ± 23; Intervention: 15–30 min (cycling/walking); Intensity: 60–80% HR peak↑(cycling); 15–30 min; 80% 6MWT (walking); Period: 3T/W; 8W	n = 17; yr: 75 ± 9; f/m: 11/6; FEV1%pred: 68 ± 19; routine nursing	6MWT; ISWT; ESWT; Borg	LG: all the indicators^b^
Daabis 2017^[25]^	—	n = 15; yr: 61 ± 8; FEV1%pred: 53.2 ± 9.5; Intervention: 30 min ET (treadmill); 30 min ST; Intensity: 75% 6MWT; Period: 3T/W, 8W	n = 15; yr: 60 ± 8; FEV1%pred: 54.6 ± 7.1 routine nursing	FEV1%pred; 6MWT; 1RM	LG/CG: except for FEV1%pred^c^, all the indicators^a^
Wootton 2017^[26]^	—	n = 62; yr: 69 ± 8; f/m: 24/38; FEV1%pred: 42 ± 15; Intervention: 30–45 min ET (walking); Intensity: 80% 6MWT, (load↑), Borg 3–4; Period: 2/3T, 8/10W	n = 39; yr: 68 ± 9; f/m: 15/24; FEV1%pred: 43 ± 15; routine nursing	6MWT; ISWT; ESWT	LG/CG: 6MWT^a^, ISWT^a^, ESWT^a^
Li 2018^[28]^	—	n = 17; yr: 66 ± 9; f/m: 5/14; FEV1%pred: 55.50 ± 16.8; Intervention: 10 min WU; 40 min ET (liuzijue); Intensity: Borg 3–4; 10 min CD; Period: 6T/W; 6M	n = 19; yr: 66 ± 9; f/m: 3/12; FEV1%pred: 58.49 ± 19.4; routine nursing	FEV1%pred; FEV1/FVC%; 6MWT	LG: FEV1%pred^a^, FEV1/FVC%p^c^, 6MWT^a^
Wu 2018^[35]^	n = 14; yr: 65 ± 11; f/m: 5/9; FEV1%pred: 59 ± 22; water temperature 32 ± 2°C Intervention: same as LG	n = 15; yr: 65 ± 8; f/m: 3/12; FEV1%pred: 55 ± 17; Intervention: 10 min WU; 40 min ET (liuzijue); 10 min CD; Intensity: Borg 4–6; Period: 2T/W; 12W	n = 16; yr: 66 ± 8; f/m: 4/12; FEV1%pred: 59 ± 17 routine nursing	FEV1%pred; FEV1/FVC%; Isokinetic muscle strength	TG: FEV1%pred^c^; FEV1/FVC%^c^; muscle strength^a^; LG/WG: function of knee joints^a^
Felcar 2018^[32]^	n = 16; yr: 69 ± 9; f/m: 6/14; FEV1%: p48 ± 17; water temperature 33 °C Intervention: same as LG	n = 16; yr: 68 ± 8; f/m: 7/9; FEV1%pred: 46 ± 14; Intervention: WU; 20–38 min ET (cycling/walking); ST; Period: 3–2T/W; 12M	—	FEV1%pred; FEV1/FVC%; 1RM; 6MWT; ISWT	TG: FEV1%pred^c^, FEV1/FVC%^c^, 1RM^b^, 6MWT^b^, ISWT^b^; LG/WG: all the indicators^c^
Gallo-Silva 2019^[29]^	n = 10; yr: 66.3 ± 6.5; f: 10; FEV1%pred: 61.0 ± 15.7; water temperature 32 °C; Intervention: 10 min WU; 20–40 min ET (aerobics/flexible exercise); 10 min CD Intensity: Borg 4–6; Period: 3T/W; 8W	—	n = 9; yr: 66.5 ± 9.5; f: 9; FEV1%pred: 60.1 ± 16.6; routine nursing	6MWT	WG/CG: 6MWT^a^
de Castro 2019^[33]^	n = 14; yr: 65 ± 8; f/m: 5/9; FEV1%pred: 51 ± 15; water temperature 33 °C; Intervention: 1 min WU; 20–38 min ET (cycling); Intensity: Borg 4–6 (cycling); Intensity: 75%6MWT/3↑ (walking); 70%1RM↑ (3 × 8); Period: 3–2T/W; 12M	n = 17; yr: 64 ± 8; f/m: 8/9; FEV1%pred: 49 ± 17; Intervention: 1 min WU; 20–38 min ET (cycling/walking); ST; Intensity: maximum individual load 60%cycling); 75%6MWT↑ (walking); 70%1RM↑ (3 × 8); Period: 3–2T/W; 12M	—	6MWT; ISWT MVIC-Q; TUG	TG: 6MWT^b^; ISWT^b^; ISWT^b^, TUG^b^; body balance^b^; LG/WG: TUG^a^

6MWT = 6-minute walking test, CD = cool down; Dyspnea = dyspnea measured at the end of the test, ET = endurance training, f/m = female/male, FEV1%pred = forced expiratory volume in the first second in percent predicted values, FEV1/FVC = ratio of forced expiratory volume in the first second to forced vital capacity, ISWT = incremental shuttle walk test, M = month, MVIC-Q = maximal voluntary isometric contraction of quadriceps, RM = repetition maximum strength test, ST = strength training, T/W = time/wk, TUG = timed up and go test, TG = training group, WU = warm-up, yr = age and year.

a Comparing are significant between groups (*P* < .05).

b Comparing are significant within group (*P* < .05).

c Comparisons are no significant between groups (*P* > .05).

### 3.3 Quality assessment

A total of 9 randomized controlled trial PEDro score results had an average of 6 points, see Table 3. Four studies^[21,30,31,34]^ scored 4 points, 3 studies^[20,25,26]^ scored 5 points, 8 studies^[22,27–29,32,33,35,37]^ scored 7 points, and 3 studies^[23,24,26]^ scored 8 points. All 18 of the studies reported baseline comparability, between-group comparisons, and point estimates and variability. Fifteen studies reported adequate follow-up, 14 studies reported random allocation, 12 studies reported concealed allocation, and 9 studies reported blind assessors, 6 studies reported intention-to-treat analysis, 2 studies reported that the blind subjects, and none of the studies reported blind therapists.

**Table 3:** PEDro scores of included studies.

Study	Inclusion criteria	Random allocation	Allocation concealment	Similar to the baseline	Participants blind	Therapists blind	Assessors blind	85% of follow-up	Intentionality analysis	Comparison between groups	Measured value	Total score 10
Bestall 2003^[20]^	Yes	1	1	1	0	0	0	0	0	1	1	5
Wadell 2004^[36]^	Yes	0	0	1	0	0	0	1	1	1	1	5
Puente-Maestu 2006^[21]^	Yes	0	0	1	0	0	0	1	0	1	1	4
Lotshaw 2007^[31]^	Yes	0	0	1	0	0	0	1	0	1	1	4
Ozdemir2010^[30]^	Yes	1	0	1	0	0	0	0	0	1	1	4
de Souto Araujo 2012^[34]^	Yes	1	0	1	0	0	0	0	0	1	1	4
Leung 2012^[27]^	Yes	1	1	1	0	0	1	1	0	1	1	7
Casey 2013^[22]^	Yes	1	1	1	0	0	1	0	1	1	1	7
McNamara 2013^[37]^	Yes	1	1	1	0	0	1	1	0	1	1	7
Wootton 2014^[23]^	Yes	1	1	1	0	0	1	1	1	1	1	8
Tsai 2017^[24]^	Yes	1	1	1	0	0	1	1	1	1	1	8
Daabis 2017^[25]^	Yes	1	0	1	0	0	0	1	0	1	1	5
Wootton 2017^[26]^	Yes	1	1	1	0	0	1	1	1	1	1	8
Li 2018^[28]^	Yes	1	1	1	0	0	1	1	0	1	1	7
Wu 2018^[35]^	Yes	1	1	1	1	0	0	1	0	1	1	7
Felcar 2018^[32]^	Yes	1	1	1	0	0	1	1	0	1	1	7
de Castro 2019^[33]^	Yes	1	1	1	0	0	1	1	0	1	1	7
Gallo-silva 2019^[29]^	Yes	1	1	1	1	0	0	1	0	1	1	7

1: Meets the PEDro scoring criteria; 0: not meet the PEDro scoring criteria.

### 3.4 Meta-analysis

#### 3.4.1 Pulmonary function

Seven studies^[21,25,28,30,32,34,35]^ evaluated the intervention effect of aerobic exercise on lung function. Compared with the CG, there was no significant difference in overall lung function of aerobic exercise in water or land (TG) (FEV1%pred, *P* = 1.02, 5 studies,^[21,25,28,30,35]^ FEV1/FVC%, *P* = .17, 4 studies^[25,28,30,35]^). Subgroup analysis showed that there was no significant difference between the LG or WG compared with the CG (Table 4).

**Table 4:** Summary of subgroup analysis.

Aerobic exercise group versus usual care. Subgroup: land versus water							
Outcome	Indicators	Subgroups	Studies/Participants	Heterogeneity	MD [95% CI]; *P*	Test for subgroup differences	Total MD [95% CI]; *P*
Lung function	FEV1%pred	Water	2/80	Tau^2^ = 0.00; *I*^2^ = 0%	2.17 [–5.31, 9.66]; .57	Chi^2^ = 0.12, df = 1 (*P* = .73), *I*^2^ = 0%	1.02 [–2.53, 4.57]; .57
		Land	4/145	Tau^2^ = 0.00; *I*^2^ = 0%	0.69 [–3.34, 4.72]; .74		
	FEV1/FVC %	Water	2/80	Tau^2^ = 0.00; *I*^2^ = 0%	0.65 [–4.21, 5.51]; .79	Chi^2^ = 0.06, df = 1 (*P* = .81), *I*^2^ = 0%	0.17 [–2.76, 3.09]; .91
Dyspnea	Borg	Land	3/115	Tau^2^ = 0.00; *I*^2^ = 0%	–0.11 [–3.78, 3.56]; .95		
		Water	2/69	Tau^2^ = 0.22; *I*^2^ = 30%	–1.09 [–2.25, 0.07]; .06	Chi^2^ = 0.57, df = 1 (*P* = .45), *I*^2^ = 0%	–0.70 [–1.12, –0.27]; .001
		Land	5/276	Tau^2^ = 0.03; *I*^2^ = 10%	–0.61 [–1.08, –0.15]; 0.01		
Exercise capacity	6MWT	Water	4/118	Tau^2^ = 0.00; *I*^2^ = 0%	80.89 [48.67, 113.11]; <.001	Chi^2^ = 2.89, df = 1 (*P* = .09), *I*^2^ = 65.4%	56.37 [32.61, 80.13]; <.001
		Land	7/380	Tau^2^ = 586.03; *I*^2^ = 45%	43.94 [16.09, 71.79]; .002		
	ISWT	Water	2/57	Tau^2^ = 0.00; *I*^2^ = 0%	27.65 [–23.30, 78.59]; .29	Chi^2^ = 0.22, df = 1 (*P* = .64), *I*^2^ = 0%	16.28 [–1.84, 34.41]; .08
		Land	8/673	Tau^2^ = 0.00; *I*^2^ = 0%	14.64 [–4.76, 34.03]; .14		
	ESWT	Water	2/60	Tau^2^ = 1174.23; *I*^2^ = 52%	339.96 [210.39, 469.53]; <.001	Chi^2^ = 1.72, df = 1 (*P* = .19), *I*^2^ = 41.9%	254.81 [166.41, 343.22]; <.001
		Land	6/361	Tau^2^ = 8405.07; *I*^2^ = 54%	228.18 [122.93, 333.42]; <.001		
Muscle strength	ULMS	Water	1/30	Not applicable	0.04 [–0.68, 0.75]; .92	Chi^2^ = 0.31, df = 1 (*P* = .58), *I*^2^ = 0%	0.20 [–0.21, 0.62]; .34
		Land	2/61	Tau^2^ = 0.00; *I*^2^ = 0%	0.28 [–0.22, 0.79]; .27		
	LLMS	Water	1/30	Not applicable	0.22 [–0.50, 0.94]; .56	Chi^2^ = 0.44, df = 1 (*P* = .51), *I*^2^ = 0%	0.00 [–0.34, 0.35]; .98
		Land	2/99	Tau^2^ = 0.00; *I*^2^ = 0%	–0.06 [–0.46, 0.33]; .76		

Note: ULMS and LLMS due to the results of different measurements using standardized mean differences (SMD). 6MWT = 6-minute walking test, CI = confidence interval, ESWT = endurance shuttle walk test, FEV1/FVC = ratio of forced expiratory volume in the first second to forced vital capacity, ISWT = incremental shuttle walk test, LLMS = lower limb muscle strength, MD = mean difference, ULMS = upper limb muscle strength.

**Figure 2:**
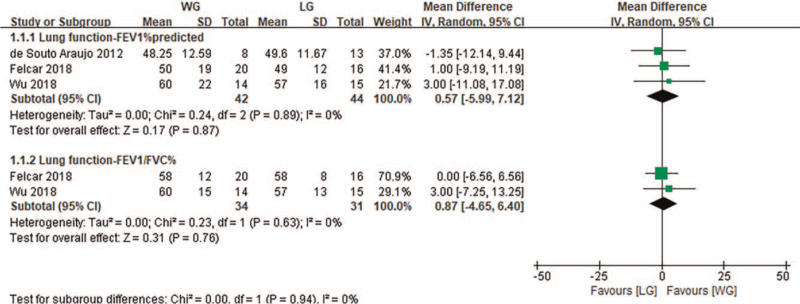
Change in lung function—water exercise group (WG) versus land exercise group (LG). CI = confidence interval, FEV1 = forced expiratory volume in the first second, FVC = forced vital capacity, SD = standard deviation.

In the comparison between water-based aerobic exercise and land-based aerobic exercise, it was found that there was no significant difference between the 2 groups after aerobic exercise intervention (FEV1%pred, *P* = .57, 3 studies,^[32,34,35]^ FEV1/FVC%, *P* = .87, 2 studies^[32,35]^, Fig. 2).

#### 3.4.2 Dyspnea

Seven studies^[21,23,24,27,30,34,37]^ used the Borg scale (0–10) to evaluate the intervention effect of aerobic exercise on dyspnea. Compared with the CG, TG significantly improved dyspnea (*P* = .001). Subgroup analysis showed significant improvement in the LG (*P* = .01), while the WG showed no significant improvement (*P* = .06, Table 4).

**Figure 3:**
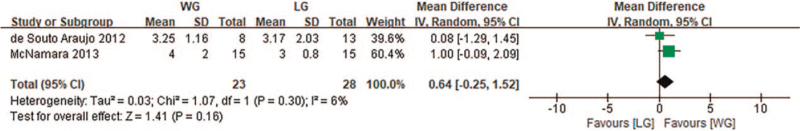
Change in dyspnea—water exercise group (WG) versus land exercise group (LG). CI = confidence interval, SD = standard deviation.

Two studies^[34,37]^ used Borg to evaluate dyspnea after water and land-based aerobic exercise intervention. Although the dyspnea score of the WG was higher than that of the LG, there was no significant difference in dyspnea between the 2 groups (*P* = .16, Fig. 3).

#### 3.4.3 Exercise capacity

Sixteen studies evaluated the effect of aerobic exercise intervention on exercise capacity, of which 9 studies^[23–26,28–30,34,37]^ used 6MWT, 8 studies^[20,22–24,26,27,36,37]^ used ISWT, and 6 studies^[23,24,26,27,36,38]^ used ESWT. Table 4 showed that compared with the CG, TG did not significantly improve ISWT (MD: 16.28, 95% CI [−1.84, 34.41], *P* = .08), but did significantly improve 6MWT (MD: 56.37, 95% CI [32.61, 80.13], *P* < .05) and ESWT (MD: 254.81, 95% CI [166.41, 343.22], *P* < .05). Subgroup analysis showed that compared with the CG, both the WG and the LG were able to significantly improve 6MWT and ESWT (*P* < .05, Table 4).

**Figure 4:**
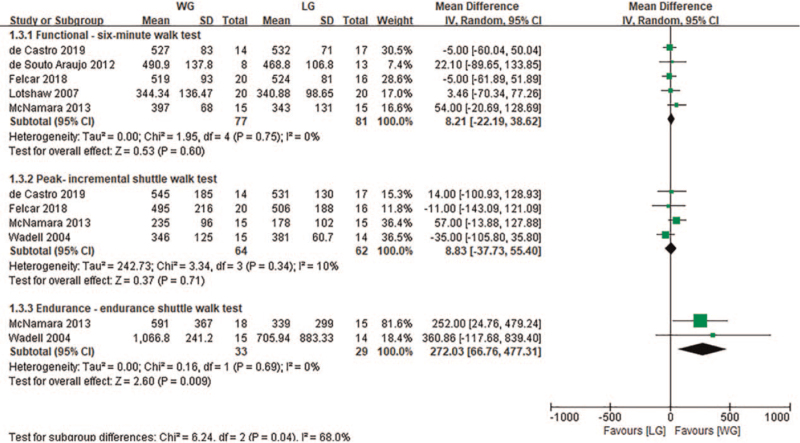
Change in exercise capacity (meter)—water exercise group (WG) versus land exercise group (LG). CI = confidence interval, SD = standard deviation.

In the comparison between water-based aerobic exercise and land-based aerobic exercise, 5 studies^[31–34,37]^ used 6MWT, while 4 studies^[32,33,36,37]^ used ISWT to assess exercise capacity. The results found that there was no significant difference between the 2 groups after the land and water-based aerobic exercise intervention (*P* = .60, *P* = .71). Additionally, 2 studies^[36,37]^ used ESWT to assess exercise capacity. The meta-analysis found that the WG showed significant improvement compared with the LG (MD: 272.03, 95% CI [66.76, 477.31], *P* = .009, Fig. 4).

#### 3.4.4 Peripheral muscle strength

Three studies^[25,27,35]^ evaluated the effect of aerobic exercise on peripheral muscle strength. Compared with the CG, TG had no significant improvement in peripheral muscle strength (ULMS, *P* = .34, LLMS, *P* = .98), and subgroup analysis showed that compared with the CG, the WG and LG were not significantly different.

**Figure 5:**
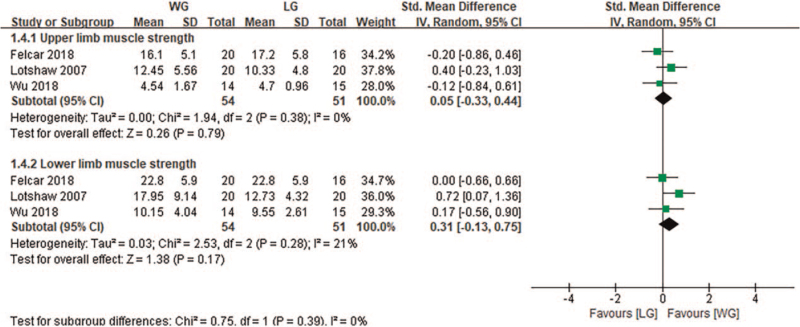
Change in muscle strength (kg)—water exercise group (WG) versus land exercise group (LG). CI = confidence interval, SD = standard deviation.

Two studies^[31,32]^ evaluated peripheral muscle strength after the water and land-based aerobic exercise intervention. Compared with the LG, there was no significant improvement in peripheral muscle strength in the WG (ULMS [SMD: 0.05 {–0.33, 0.44}, *P* = .79] and LLMS [SMD: 0.31, 95% CI {–0.13, 0.75}, *P* = .17], Fig. 5).

#### 3.4.5 Adverse events

**Figure 6:**
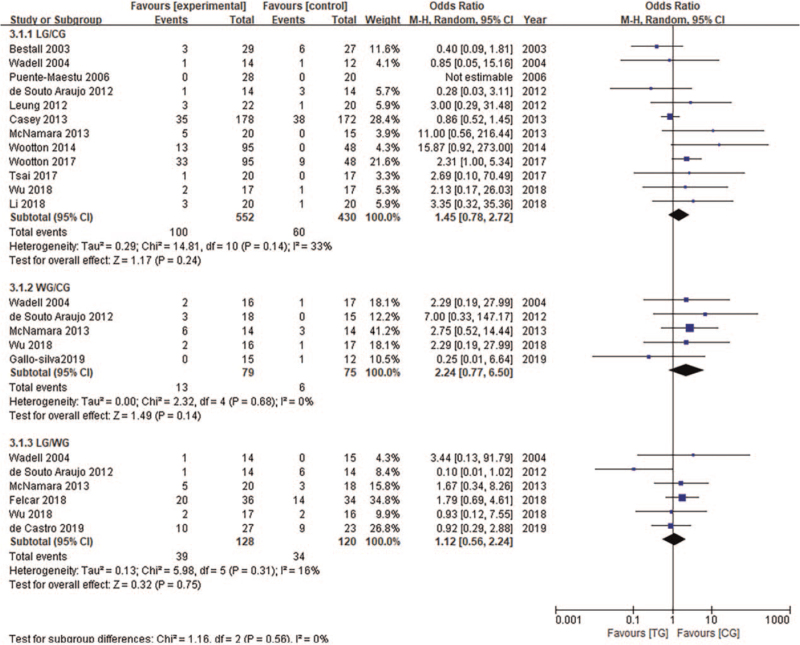
Adverse events were compared among the 3 groups. CI = confidence interval, SD = standard deviation.

Although the severity of COPD differed between studies, the results reveal that (Fig. 6) there was no significant difference in the dropout rates among COPD patients in the different intervention environments (CG, LG, WG). Six studies reported that a total of 37 patients withdrew due to health problems (LG = 21, WG = 16) from both land and water-based aerobic exercise groups. These health problems included deterioration due to the disease, cancer, orthopedics, and diabetes complications, vascular disease, and diarrhea. Eighteen patients withdrew because of lack of interest (LG = 14, WG = 4).

## 4 Discussion

The cornerstone of PR is aerobic exercise, and this systematic review shows that the functional exercise capacity, exercise endurance, and dyspnea of COPD patients show significant improvement after aerobic exercise. Whether aerobic exercise is carried out on water or land, the increased adaptability in patients’ physical activity and the improvement in aerobic capacity are similar. In addition, compared with land-based exercise, aerobic exercise in water can significantly improve exercise endurance. According to reports, aerobic exercise improves the body's oxidation capacity, improves the vital capacity of COPD patients, reduces dynamic hyperinflation, and enhances cardiopulmonary adaptability.^[17,38]^ The results of this study found that there was no significant statistical significance in lung function after aerobic exercise. The reason for the difference in results is not only affected by the baseline level of included patients, but also by the length of the intervention period. When we analyzed 2 studies^[28,35]^ with intervention periods longer than 8 weeks, a trend toward lung function improvement was observed in the data (FEV1%pred: 2.05 [–4.75, 8.86], FEV1/FVC%: 3.00 [–7.43, 13.43]). It is speculated that long-term aerobic exercise intervention may have positive physiological effects. Previous studies found that water exercise, as compared with land exercise, had an additional positive role in maintaining and improving lung function (especially FEV1).^[17]^

It may be that the combination of the hydrostatic pressure and water temperature induces an increase in cardiac output,^[39]^ a decrease in sputum viscosity, and an increase in respiratory frequency, thus increasing the rate of gas exchange in the lungs. The results of this study show that water-based aerobic exercise has beneficial effects on patients’ lung function, but there is no significant difference compared with land-based aerobic exercise. Therefore, more studies are needed in the future to determine the effect of water-based aerobics on lung function in COPD patients and the improvement attributable to long-term intervention.

Dyspnea is one of the main discomforts of COPD patients. Progressive dyspnea can lead to fatigue,^[40]^ prevent physical exercise, and reduce the functional level of patients.^[9]^ Studies found that after aerobic exercise intervention combined with respiratory muscle and auxiliary muscle stretching,^[41,42]^ the dyspnea of COPD patients decreased, the respiratory efficiency needed for ventilation increased,^[18]^ and upper limb fatigue was significantly improved.^[42]^ We observed that the dyspnea level of patients in the aerobic training group decreased significantly by 0.70 (95% CI:–1.12, –0.27). This decline may be due to the inclusion of studies involving not only movements of large muscle groups, but also the intervention of respiratory muscle auxiliary muscles. During exercise, passive chest muscle stretching and rib expansion will increase the burden on the diaphragm, which is equivalent to the load training of respiratory muscle groups, which can improve respiratory muscle strength and exercise tolerance.^[17]^ In addition, we cannot completely rule out another explanation, which is that aerobic training has a therapeutic effect on lung function, because we noticed that FEV1%pred and FEV1/FVC%, increased by 2.05 and 3.00, respectively, after aerobic training in this study. Therefore, aerobic training may relieve hyperinflation by improving lung function and respiratory muscle function, thus improving dyspnea in COPD patients.

**Figure 7:**
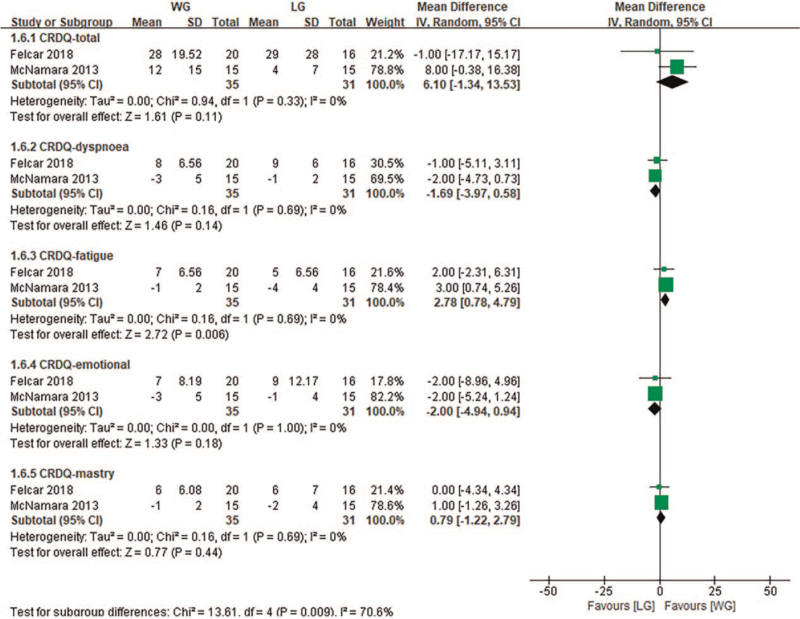
Change in quality of life scores—water exercise group (WG) versus land exercise group (LG). CI = confidence interval, CRDQ = chronic respiratory disease questionnaire, SD = standard deviation.

Previous studies have shown that under the same exercise intensity, compared with land-based aerobic exercise, the parameters of heart rate and blood lactic acid in water decreased, and dyspnea and fatigue perception were also significantly improved.^[43]^ This study found similar results, specifically, that dyspnea between the 2 groups was similar at the same or relative exercise intensity. However, chronic respiratory disease questionnaire fatigue perception was significantly improved after water-based aerobic exercise (Fig. 7). Given these, participants may achieve or even exceed the required exercise intensity through less exercise time in a fatigue-relieving water environment, which is especially important for weak and elderly exercisers.

A variety of testing methods can be used to assess the exercise capacity of COPD patients. Among them, 6MWT can reflect the functional exercise capacity and quality of life,^[44]^ ISWT can effectively and reliably reflect the maximum exercise capacity of patients with self-limited symptoms,^[45]^ and ESWT reflects the exercise endurance of COPD patients, and is also sensitive to changes in treatment and intervention in patients with severe COPD.^[46]^ This study found that after aerobic training, the increasement of 6MWT and ESWT exceeded the minimum clinically important difference reported in the literature.^[44]^ However, in studies based on aerobic exercise in different water and land environments, exercise capacity showed different performance in the outcome index.^[8,19]^ For example, the results of Shead and Aswegen^[18]^ and McNamara et al^[16]^ found that ESWT was significantly increased in both water and land groups, but the results of ISWT were particularly different. These studies show contradictory results, which may be due to the included study, the frequency, time and content of the intervention, and the severity of the disease. In this study, there was no significant improvement in ISWT between the aerobic exercise group and the control group, or between the water-based aerobic exercise and the land-based aerobic exercise groups. In addition to the above factors, another reason may be that the heart rate increased linearly with the increasing workload during the ISWT, and the dyspnea score increased linearly in the later stage of ISWT,^[47]^ causing more cardiovascular and dyspnea reactions on the ISWT than the 6MWT.^[48]^ However, this study shows a significant difference between water and land-based aerobic exercise on the ESWT. The ESWT in the water was significantly increased by 254.81 m (95% CI: 166.41, 343.22), and chronic respiratory disease questionnaire fatigue perception was decreased significantly, suggesting that an increase in exercise endurance and increase in long-term endurance walking distance were associated with general and activity fatigue.^[49]^ Therefore, based on the improvement of exercise endurance and relieving fatigue, COPD patients are encouraged to perform the aerobic exercise in water. This is especially important for exercisers with severe COPD who are unable to stand for a long time, or who have exercise intolerance.

Analysis of relationships between exercise capacity and other outcome indexes of PR revealed that the increase of exercise tolerance time is related to the increase of forced inspiratory volume, the decrease of respiratory frequency, and the decrease of fatigue perception.^[44,45,49,50]^ Previously, studies of exercise capacity in COPD showed that the exercise capacity of COPD patients is also related to muscle strength.^[46,51]^ In this study, the exercise capacity of patients was improved after aerobic training, but there was no significant change in peripheral muscle strength. Perhaps aerobic training increases muscle oxidation, rather than the cross-sectional area and muscle mass, for which resistance training is more effective.^[52]^ This suggests that aerobic exercise may not impact immediate muscle strength, but it may improve the muscle oxidation ability of COPD patients, which has a long-term effect on exercise capacity. Studies have shown that water exercise is beneficial to pain,^[53]^ physical function,^[54]^ and LLMS^[55]^ in patients with musculoskeletal diseases. In this study, there was no statistical difference in the peripheral muscle strength of patients who underwent water and land-based aerobic exercise (ULMS 0.05 kg [95% CI: –0.33, 0.44], LLMS 0.31 kg [95% CI: –0.13, 0.75]). Only one^[37]^ study of patients with skeletal muscle complications found a superior rehabilitation effect for LLMS in patients who underwent water-based exercise intervention compared with patients who performed land-based exercise. In the subgroup analysis, the authors also confirmed that obese patients with COPD not only lost weight but also showed improved exercise capacity and quality of life after water-based exercise.^[56]^ These physiological improvements may be related to the general low peripheral muscle strength and physical activity of this group.

At the same time, the exercise intensity in the water cannot be quantified, and the lack of load leads to low intensity in the individual strength training. Therefore, with the advance of PR, the peripheral muscle strength of patients is unlikely to improve significantly. More studies that water aerobic exercise is needed in the future, especially to further determine whether exercise intensity and COPD patients’ baseline peripheral muscle strength are important factors affecting muscle strength improvement. Currently, there is great controversy about the feasibility and acceptability of water-based exercise as a form of PR in patients with COPD. Hydrostatic pressure may lead to an increase in chest pressure, resulting in respiratory limitation.^[57]^ In addition, some irritating gases in the pool may also cause asthma and allergies, aggravating symptoms, such as cough, wheezing, and dyspnea.^[58]^ However, an increasing number of studies have reported that water-based exercise increases venous drainage and cardiopulmonary load,^[59]^ and brings pleasure, novelty, and excitement to patients.^[19]^ Therefore, water aerobics can be applied to COPD patients as a safe and effective intervention to stimulate sports interest.

This type of systematic evaluation with meta-analysis has some limitations. First, although the inclusion of 18 randomized controlled trials involved 1311 cases, the sample size of most studies was relatively small. Additionally, there was heterogeneity in the sample and methodology of the included studies. Although the contents of the exercise programs and the outcomes in the studies were roughly similar, the intensity and duration of the exercise programs and evaluation methods varied greatly. The measurement methods and units of the outcomes were not unified, which may have led to bias after the unit conversion that is possible factors leading to clinical heterogeneity and biases in the interpretation of the data. Finally, concerning the quality evaluation included in the studies, the allocation concealment was not implemented in 6 studies, and a lack of blinding practices (of the participants, outcome assessors, and therapists) were also significant limitations.

## 5 Conclusion

Aerobic exercise can improve dyspnea and enhance both functional exercise capacity and exercise endurance in COPD patients. Whether aerobic exercise is carried out in water or on land, the adaptability of patients’ physical activity and the improvement of aerobic capacity are similar. However, the improvement in exercise endurance after aerobic exercise in water is more prominent than the improvement of land-based exercise. The properties of water make COPD patients need more lung ventilation and more energy expenditure during exercise, which may trigger relatively greater cardiopulmonary and/or neuromuscular effects, then induce better therapeutic effects. Secondly, it is observed that there is a greater difference in fatigue perception of this study. Water aerobic exercise caused a significant improvement in interfering with fatigue perception. Finally, no adverse events were found under different external environment, water aerobic exercise is as safety as land aerobic exercise. Therefore, PR program should try to combine with water environment in the future. We suggest that water aerobic exercise is more suitable for those patients who are unable to stand for an extended time and have skeletal muscle complications.

However, existing researches are insufficient in quantizing the water aerobic exercise intensity, and there are few researches conducted with progressive programs. In the future research, professional physician or therapists should focus on the specific effects of water and land aerobic exercise, determine and compare the follow-up effects in different time points, conduct gradual progressive exercise program on the basis of individual characteristics. At the same time, future research should focus on determining the respective specific efficacies of water and land-based aerobic exercise, and on prolonging the intervention and follow-up time, to further explore the rehabilitation effects of water and land-based aerobic exercise in patients with COPD.

In summary, water aerobic exercise can provide COPD patients with a low-cost and effective group therapy, which should be considered in public relations project of the family, community or hospital.

## Acknowledgments

The authors thank Ting Wang and Yongdi Zou for their excellent work in this study, we also thank LetPub (http://www.letpub.com) for its linguistic assistance during the preparation of this manuscript.

## Author contributions

**Conceptualization:** Haixia Chen, Weibing Wu, Jihong Wang.

**Data curation:** Haixia Chen, Ling Li, Zhengrong Wang.

**Investigation:** Haixia Chen, Zhengrong Wang.

**Methodology:** Ling Li.

**Supervision:** Peijun Li.

**Writing – original draft:** Haixia Chen.

**Writing – review & editing:** Peijun Li, Weibing Wu, Jihong Wang.
